# Recent advances in malaria genomics and epigenomics

**DOI:** 10.1186/s13073-016-0343-7

**Published:** 2016-09-07

**Authors:** Sebastian Kirchner, B. Joanne Power, Andrew P. Waters

**Affiliations:** Wellcome Trust Centre for Molecular Parasitology, Institute of Infection, Immunity and Inflammation, College of Medical Veterinary & Life Sciences, University of Glasgow, Glasgow, G12 8TA UK

## Abstract

Malaria continues to impose a significant disease burden on low- and middle-income countries in the tropics. However, revolutionary progress over the last 3 years in nucleic acid sequencing, reverse genetics, and post-genome analyses has generated step changes in our understanding of malaria parasite (*Plasmodium* spp.) biology and its interactions with its host and vector. Driven by the availability of vast amounts of genome sequence data from *Plasmodium* species strains, relevant human populations of different ethnicities, and mosquito vectors, researchers can consider any biological component of the malarial process in isolation or in the interactive setting that is infection. In particular, considerable progress has been made in the area of population genomics, with *Plasmodium falciparum* serving as a highly relevant model. Such studies have demonstrated that genome evolution under strong selective pressure can be detected. These data, combined with reverse genetics, have enabled the identification of the region of the *P. falciparum* genome that is under selective pressure and the confirmation of the functionality of the mutations in the *kelch13* gene that accompany resistance to the major frontline antimalarial, artemisinin. Furthermore, the central role of epigenetic regulation of gene expression and antigenic variation and developmental fate in *P. falciparum* is becoming ever clearer. This review summarizes recent exciting discoveries that genome technologies have enabled in malaria research and highlights some of their applications to healthcare. The knowledge gained will help to develop surveillance approaches for the emergence or spread of drug resistance and to identify new targets for the development of antimalarial drugs and perhaps vaccines.

## Background

Malaria, caused by unicellular protozoan *Plasmodium* spp. parasites, is an ancient disease and remains a major threat to human health and wellbeing. Five species of *Plasmodium* are currently recognized as causing human malaria, of which the most lethal is *P. falciparum* (Pf). In 2015, the World Health Organization estimated that the maximal annual burden imposed by malaria, whilst decreasing, still stands at 214 million (range 149–303 million) cases that result in 438,000 (range 236,000–623,000) deaths [1]. Drug resistance to frontline antimalarials continues to arise and spread, exacerbated by slow progress in the introduction of alternatives. Properly efficacious vaccines remain a hope, not a likelihood. Against this background, genome-based research on malaria seeks to provide new avenues for therapeutic or prophylactic development based upon biological insights such as the identification of new drug targets and vaccine candidates.

**Fig. 1:**
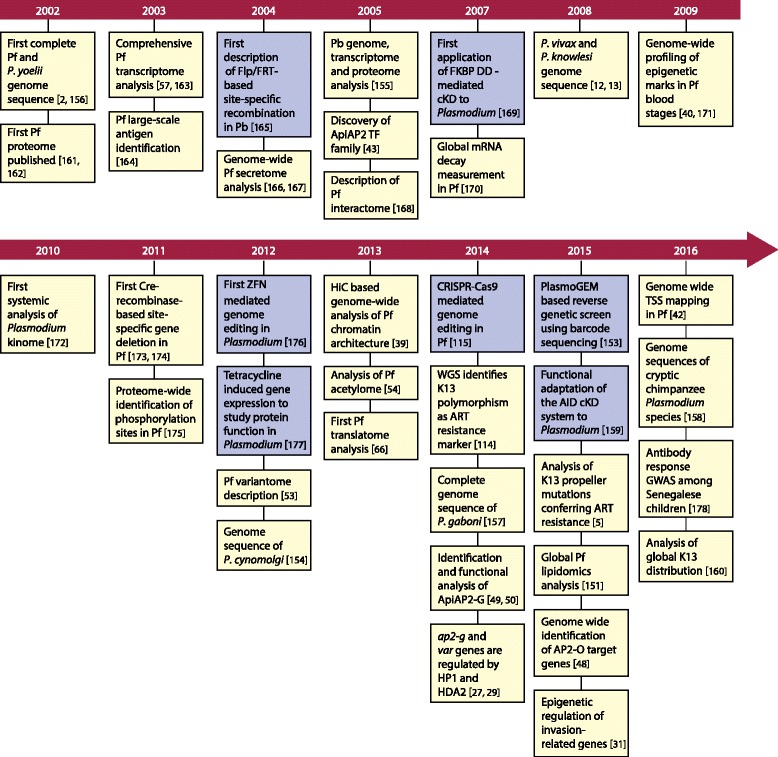
Major advances in omics-related fields. This figure highlights landmark studies providing key insights into parasite makeup, development, and pathogenesis (*yellow boxes*) as well as crucial technical advances (*blue boxes*) since the first *Plasmodium* genomes were published in 2002 [2, 5, 12, 13, 27, 29, 31, 39, 40, 42, 43, 48–50, 53, 54, 57, 66, 114, 115, 151, 153–178]. *AID* auxin-inducible degron, *ART* artemisinin, *cKD* conditional knockdown, *CRISPR* clustered regularly interspaced short palindromic repeats, *DD* destabilization domain, *K13* kelch13, *Pb P. berghei*, *Pf P. falciparum*, *TSS* transcription start site, *TF* transcription factor, *ZNF* zinc finger nuclease

The landmark of the completion of the genome sequence of a laboratory strain of Pf was achieved over a decade ago [2] (Fig. 1). This has since been accompanied, thanks to plummeting costs and advances in next-generation sequencing (NGS) technologies, by the whole-genome sequencing (WGS) of a wide range of species representing all the major clades of the genus, although the genomes of all known human infectious *Plasmodium* species remain to be sequenced [3]. However, the combination of NGS and WGS has enabled the development of innovative large-scale genomic studies, for example, for genomic epidemiology [4]. Such population genomics, fueled by collaborative consortia (for example, the Malaria Genomic Epidemiology Network (MalariaGEN; http://www.malariagen.net), have allowed the dynamics of global and local population structures to be assessed and adaptive change in parasite genomes to be monitored in response to threats, such as artemisinin (ART). This is especially true for single-nucleotide polymorphisms (SNPs), and while other aspects of genome variation (such as indels and copy number variation) might currently lag behind, the gaps in the database are known and are firmly in the sights of researchers.

The template *Plasmodium* genomes have provided the substrate for the application of an explosion of other post-genome survey technologies that have been largely exclusively applied to Pf, such as transcriptomics, proteomics, metabolomics, and lipidomics, and that map the general and stage-specific characteristics of malaria parasites. These data are warehoused in expensive but critical community web sites such as PlasmoDB (http://www.Plasmodb.org). This in turn has been exploited by ever improving forward and reverse genetic capabilities to assign function to genes, steadily reducing the >60 % of genes of unknown function that were originally catalogued [2]. Advances that will be highlighted in this review include: the unraveling of the molecular mechanisms of parasite resistance to ART; the functional identification of some of the histone-modifying enzymes that write the epigenetic code (such as Pf histone deacetylase 2 (PfHDA2)) and the proteins that read it (such as Pf heterochromatin protein 1 (PfHP1)) that, with others (such as RNaseII), play a significant role in the regulation of antigenic variation and commitment to sexual development.

Furthermore, the genomes of the host and of a growing number of mosquito vectors have been characterized in both increasing number and depth, permitting meta-analyses of these genomes in combination with *Plasmodium* infection. These studies have revealed important loci associated with resistance to the malaria parasite in the host and vector, respectively [5, 6], and indicate the genomic hotspots in the genetic arms race that malaria has stimulated.

We also review the recent advances in this very active area of malaria genomics and control of gene expression and emphasize any benefits that these advances may have for the development of therapies and interventions (Table 1).

**Table 1:** Key advances from recent omics studies

Research area	Key findings	Reference(s)
Genome sequencing	WGS and refinement of genome sequences of primate and rodent infectious *Plasmodium* species	[13, 154–157, 179]
	Genome sequence of cryptic chimpanzee *Plasmodium* species	[158]
Gene expression regulation	Discovery of AP2-G as master regulator of gametocytogenesis in Pf and Pb	[49, 50]
	AP2-G2 functions as a transcription regulator driving gametocyte development	[51]
	Functional analysis of Pf lifecycle dynamics using transcriptional profiling	[180]
	Genome-wide TSS mapping in Pf	[42]
	Acetylome analysis reveals post-translational PTM of ApiAP2 transcription factors	[55]
	Genome-wide analysis of AP2-O target genes	[48]
Post-transcriptional/translational control	Genome-wide analysis reveals features of the Pf translatome	[59, 66]
	Analysis of the Pb maternal repressome	[64]
Epigenetic control	SETvs functions as a regulator of *var* gene silencing in Pf	[98]
	Involvement of HP1 in clonal variant gene expression and sexual development	[29]
	HDA2 mediates *var* gene silencing and sexual commitment in Pf	[27]
	BDP1 regulates expression of invasion-related genes	[31]
	Profiling of histone PTMs in Pf	[22]
Gene silencing/editing	First description of the use of ZFN and CRISPR-Cas9 genome editing in *Plasmodium*	[115, 116, 140]
	Functional adaptation of an AID-based conditional knockdown system in Pb	[159]
Antibiotic resistance	Analysis of KELCH13-propellermutations in *Plasmodium* field isolates confirms relation to artemisinin resistance	[115, 116]
	Identification of UPR pathway involvement in *Plasmodium* artemisinin resistance	[123, 126]
Genome structure	First profiling of genome-wide chromosome interactions	[39]
	Strong correlation between spatial genome structure and gene expression	[37]
ncRNAs	PfRNaseII exonuclease is involved in group A *var* gene silencing	[84]
	Genome-wide search revealed over 1000 novel ncRNAs in Pf	[181]
	Strand-specific RNA sequencing reveals widespread transcription of antisense RNAs in Pf	[16–18]
	Antisense lncRNAs regulate expression of cognate *var* genes in Pf	[87]
Virulence/transmission	Vector transmission of *P. chabaudi chabaudi* impacts on parasite virulence	[182]
	Transcriptome changes during early phase of mosquito infection	[183]
Development	Global phosphoproteome analysis identified PKG as major intracellular Ca^2+^ level regulator	[184]
	PKG regulates *Plasmodium* egress and evasion	[185]
	Systematic subtractive bioinformatic analysis identified sex-specific stage V gametocyte protein marker	[186]
	Protein phosphatases are key regulators of *Plasmodium* development	[187]
Population genomics	Identification of a novel malaria resistance locus through GWAS in African children	[5]
	Genomic epidemiology study revels global KELCH13 mutation distribution	[160]
Metablomics	Lipid metabolism analysis in Pf	[151]

*AID* auxin-inducible degron, *CRISPR* clustered regularly interspaced short palindromic repeats, *GWAS* genome-wide association study, *lncRNA* long non-coding RNA, *ncRNA* non-coding RNA, *Pb P. berghei*, *Pf P. falciparum*, *PTM* post-translational modification, *SNP* single-nucleotide polymorphism, *TSS* transcription start site, *UPR* unfolded protein response, *WGS* whole-genome sequencing, *ZFN* zinc finger nuclease

## Human genomics

The infrastructure required to effectively collect, collate, and analyze large genomes for epidemiological studies (that is, genome-wide association studies (GWAS)) is so costly that it is best achieved in consortia. These can work at such a scale that analyses are powered to a degree that GWAS findings become more certain and the global context of the effect of, for example, human genetics on susceptibility to malaria is more reliably resolved. The African Genome Variation Project recognizes the significant diversity of ethnicities and, therefore, genotypes and, through WGS, imputation, and SNP mapping, seeks to build a database through which disease incidence and outcome can be reliably associated with haplotypes [7]. Already, such wider analyses have confirmed SNP associations with five well-known traits, including hemoglobinopathies and glucose-6-phosphate dehydrogenase (G6PD) deficiency, but have refuted 22 others that had been linked by smaller-scale studies [8]. This study also showed opposing effects of G6PD on different fatal consequences of malaria infection, revealing a hitherto unsuspected complexity of associations. Ongoing analyses have revealed new, although unsurprising, examples of haplotypes of loci associated with protection from severe malaria, such as the glycophorin locus on human chromosome 4 [8, 9].

## Vector genomics

In Africa, malaria is mainly transmitted by female *Anopheles gambiae* (Ag) mosquitoes. Approaches to understanding the role of Ag mosquito genomics in malaria transmission have been similar to those of the African Genome Variation Project. Thus, the Ag1000G project (https://www.malariagen.net/projects/ag1000g) involves 35 working groups that have sampled Ag mosquitos from 13 malaria endemic countries and that aim to establish the levels of Ag genome diversity, establish population structures, and link these to the ecology of disease transmission. The *Anopheles* vector genome is very dynamic. Comparative vector genomics has revealed rapid gene gain and loss compared with *Drosophila* and significant intragenus diversity and mixing in genes involved in both insecticide resistance and antimalarial immunity [10, 11]. The nature and extent of such diversity precludes the application of classic GWAS approaches and a novel approach of phenotype-driven, pooled sequencing coupled with linkage mapping in carefully selected founder colonies has been used to map vector phenotypes. This study recently revealed *TOLL11* as a gene that protects African mosquitoes against Pf infection [6].

## Parasite genomics

Full genome sequences are now available for many strains of Pf [2], *Plasmodium vivax* [12], and *Plasmodium knowlesi* [13] among the human infectious parasites. Primate and rodent infectious species that are frequently used as model parasites have also been sequenced and include *Plasmodium berghei* (Pb), *Plasmodium cynomolgi, Plasmodium chabaudi* and *Plasmodium yoelii* [14]. Recently, the genomes of seven further primate infectious species have become available, demonstrating the close relationship between Pf and chimpanzee infectious species [15]. The typical *Plasmodium* genome consists of 14 linear chromosomes of aggregate size of approximately 22 megabases encoding >5000 protein-coding genes. The core, conserved genome of about 4800 such genes occupies the central chromosomal regions whilst the multi-gene families (at least some of which are associated with antigenic variation) are largely distributed to the subtelomeric regions. Non-coding RNA (ncRNA) genes [16] and antisense transcription [17, 18] are being catalogued in Pf but this catalogue probably remains incomplete as only blood-stage parasites have been seriously investigated in this regard and ncRNAs remain largely of unknown significance.

One key feature of Pf is its evolution in the face of human-imposed selection pressures in the form of drugs and potentially vaccines. Such pressure has consistently resulted in the emergence of drug-resistant parasites. There is a huge potential global reservoir of genome variation upon which selection may act. In an initial analysis of 227 parasite samples collected at six different locations in Africa, Asia, and Oceania, MalariaGEN, the Oxford-based genomic epidemiology network, identified more than 86,000 exonic SNPs. This initial SNP catalogue is described in detail by Manske and colleagues [19]. Currently (27 July 2016), the MalariaGEN database states that for the Pf Community Project, it has data on 3488 samples from 43 separate locations in 23 countries and the number of high-quality, filtered exonic SNPs has increased to more than 900,000. All of this variation is diversity, which in turn can be selected for fitter and perhaps more deadly parasites. Modern NGS and WGS have enabled comparative and population genomics approaches that have been used to reveal important features of emerging parasite populations, for example, in response to drugs.

### Parasite development and pathogenesis

**Fig. 2:**
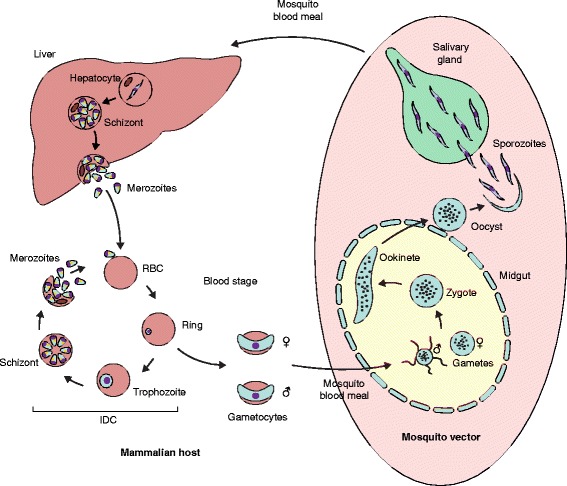
*Plasmodium* life cycle. After a mosquito bite, malaria parasites are deposited into the host’s skin and within minutes are carried via the bloodstream into the liver, where through asexual proliferation within the hepatocytes tens of thousands of merozoites are produced. Following hepatocyte rupture, merozoites are released into the bloodstream where they can invade the host’s red blood cells (*RBC*), leading to the initiation of the intra-erythrocytic development cycle (*IDC*). During the IDC (lasting about 48–72 h in human and about 24 h in rodent malaria parasites), *Plasmodium* parasites multiply asexually through the completion of several morphologically distinct stages within the RBCs. After RBC invasion, malaria parasites develop via the ring and trophozoite stage into schizonts, each containing a species-specific number of merozoites (typically 10–30). Upon schizont rupture, merozoites are released into the bloodstream, where they can invade new RBCs and initiate a new IDC. However, a small fraction of ring-stage parasites sporadically differentiate into male or female gametocytes, which are responsible for initiating transmission back to the mosquito. Through another mosquito blood meal gametocytes are taken up into the mosquito midgut where they are activated and form male (eight per gametocyte) and female (one) gametes. Following fertilization, the zygote undergoes meiosis (and therefore true sexual recombination) and develops into a motile, tetraploid ookinete that traverses the midgut and forms an oocyst. Via another round of asexual proliferation inside the oocyst several thousands of new haploid sporozoites are generated that, upon their release, colonize the mosquito salivary glands, poised to initiate a new infection of another mammalian host

Within their mammalian host and mosquito vector *Plasmodium* parasites complete a remarkable lifecycle, alternating between asexual and sexual replication (Fig. 2). Throughout the *Plasmodium* lifecycle, regulation of gene expression is orchestrated by a variety of mechanisms, including epigenetic, transcriptional, post-transcriptional, and translational control of gene expression. Owing to the absence of most canonical eukaryotic transcription factors in the *Plasmodium* genome [2], epigenetic control has long been recognized to play an important role in gene expression regulation.

**Fig. 3:**
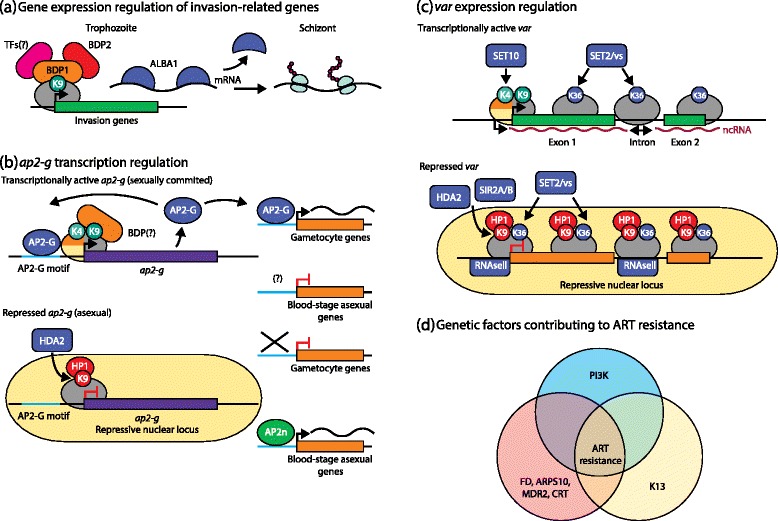
Malaria parasite genomic components involved in pathogenesis. **a** The expression of invasion-related genes is regulated through epigenetic and post-transcriptional mechanisms. Bromodomain protein 2 (*BDP2*) binds to H3K9ac marks within the promoter region of genes associated with red blood cell (RBC) invasion (as well as other gene families not depicted here [31]), enabling their transcription. This is likely achieved through the recruitment of BDP1 and transcription factors (*TFs*) of the ApiAP2 family. Following transcription during the trophozoite stage, mRNAs encoding invasion-related proteins are bound by ALBA1 functioning as translation repressor. After progression to the schizont stage, ALBA1 is released, allowing the timely synthesis of proteins required for merozoite invasion of RBCs. **b** Experimental findings either directly from studies on *ap2-g* or from epigenetically regulated *var* genes are suggestive of an epigenetically controlled mechanism regulating *ap2-g* transcription. In sexually committed parasites, *ap2-g* is characterized by H3K4me2/3 and H3K9ac histone marks and most likely contains histone variants H2A.Z and H2B.Z located in its promoter region. BDPs are believed to bind to H3K9ac, facilitating *ap2-g* transcription. ApiAP2-G drives expression of genes required for sexual development through binding to a 6/8-mer upstream DNA motif. *ap2-g* expression itself is believed to be multiplied through an autoregulatory feedback loop where ApiAP2-G binds to its own promoter that also contains ApiAP2-G motifs. In asexual blood-stage parasites, *ap2-g* is transcriptionally silenced by heterochromatin protein 1 (*HP1*) binding to H3K9me3 histone marks (located in repressive loci in the nuclear periphery). Histone deacetylase 2 (*HDA2*) catalyzes the removal of H3K9ac from active *ap2-g*, facilitating *ap2-g* silencing. **c** Monoallelic expression of one of the approximately 60 members of the erythrocyte membrane protein 1 (EMP1)-encoding *var* genes is regulated through epigenetic silencing of all but one *var* gene copy. The active *var* is marked by euchromatin post-translational modifications H3K4me2/3 and H3K9ac and histone variants H2A.Z/H2B.Z located in its promoter region, as well as H3K36me3 covering the whole *var* gene body but absent from the promoter region. Transcription of noncoding RNAs associated with the active *var* gene is facilitated by upstream as well as intronic promoters. All other silenced *var* genes cluster into perinuclear repressive loci and are characterized by HP1 binding to H3K9me3 marks. *var* gene silencing also involves SET2/vs-dependent placing of H3K36me3 histone marks in promoter regions and is marked by the absence of non-coding RNAs, likely safeguarded through RNaseII exonuclease activity. In addition, other histone code modulators such as HDA2, SET10, and SIR2A/B are likely involved in epigenetic *var* gene regulation. **d** Mutations in *kelch13* (K13) were found to be the major contributors to artemisinin (*ART*) resistance identified in drug-resistant parasites in the laboratory as well as in field isolates. *kelch13* mutations appear to arise in a complex array of background mutations (that is, mutations in genes encoding ferredoxin (*FD*), apicomplast ribosomal protein S10 (*ARPS10*), multidrug resistance protein 2 (*MDR2*), and chloroquine resistance transporter (*CRT*)), not yet detected in African parasites. In addition, elevated phosphatidylinositol-3-kinase (*PI3K*) levels have been observed in ART-resistant parasites and PI3K signaling has been implicated to impact on the unfolded protein response observed in ART-resistant parasites. H2A.Z/H2B.Z, *orange*/*yellow-paired quarter circles*; H3K4me2/3, *light green circles*; H3K9ac, *dark green circles*; H3K9me3, *red circles*; H3K36me3, *blue circles*; canonical nucleosomes, *grey globes*; ApiAP2-G binding motif; *light blue line*; ncRNAs, *wobbly red lines*; mRNAs, *wobbly black lines. AP2n* other TFs belonging to the ApiAP2 DNA binding protein family, *ncRNA* non-coding RNA, *TFs* transcription factors

Epigenetics lies at the very heart of gene expression, regulating access of the transcriptional machinery to chromatin [20] via (1) post-translational modifications (PTMs) of histones, (2) nucleosome occupancy, and (3) global chromatin architecture. In the past decade, various histone PTMs have been identified throughout the *Plasmodium* lifecycle (reviewed in [21]) and the existing catalog of modifications in Pf was recently extended to 232 distinct PTMs, 88 unique to *Plasmodium* [22]. The majority of detected PTMs show dynamic changes across the intra-erythrocytic development cycle (IDC), likely mirroring changes within chromatin organization linked to its transcriptional status. Methylation and acetylation of N-terminal histone tails are by far the most studied regulatory PTMs, linked either to a transcriptionally active chromatin structure (that is, euchromatin) or to transcriptionally inert heterochromatin. In Pf, various genes encoding putative epigenetic modulators (that is, proteins catalyzing either the addition or removal of histone PTM marks) have been identified [23], but only a few have been subjected to more detailed investigation [24, 25]. Many of the histone modifiers are essential for *Plasmodium* development, making them a promising target for antimalarial drugs [26]. In Pf, conditional knockdown of HDA2, a histone lysine deacetylase (HDAC) catalyzing the removal of acetyl groups from acetylated histone 3 lysine 9 (H3K9ac), resulted in elevated H3K9ac levels in previously defined heterochromatin regions [27]. H3K9ac is an epigenetic mark associated with transcriptionally active euchromatin [28] and HDA2 depletion resulted in the transcriptional activation of genes located in heterochromatin regions, leading to impaired asexual growth and an increased gametocyte conversion [27]. Interestingly, genes found to be dysregulated by HDA2 knockdown are also known to be associated with HP1, a key epigenetic player binding to tri-methylated H3K9 (H3K9me3), linked to transcriptionally repressed chromatin. Strikingly, conditional knockdown of PfHP1 recapitulated, to a much greater extent, the phenotype observed in HDA2-knockdown mutants [29]. HP1 is believed to act as a recruitment platform for histone lysine methyltransferases (HKMTs), required for maintenance and spreading of H3K9me3 marks [30], which is consistent with the reduction of H3K9me3 observed in HP1 knockdown cells [29]. In addition, bromodomain protein 1 (BDP1) was found to bind to H3K9ac and H3K14ac marks within transcription start sites (TSSs) in Pf, among them predominantly invasion-related genes (Fig. 3a), and BDP1-knockdown parasites consistently failed to invade new erythrocytes. BDP1 also appears to act as a recruitment platform for other effector proteins such as BDP2 and members of the apicomplexan AP2 (ApiAP2) transcription factor family [31].

In addition to histone PTMs, nucleosome organization plays a critical role in gene expression regulation in *Plasmodium*. In general, heterochromatin is substantially enriched in nucleosomes compared with euchromatin [32] and active promoters and intergenic regions in Pf show markedly reduced nucleosome occupancy [33]. In addition, common transcript features such as TSSs, transcription termination sites, and splice donor/acceptor sites show clearly distinguishable nucleosome positioning in Pf [34], but previously described dynamic changes in nucleosome positioning [32] appeared to be mostly restricted to TSSs during the IDC [34]. Uniquely in *Plasmodium* spp., canonical histones in intergenic regions are replaced by histone variant H2A.Z [28], which, in concert with the apicomplexan-specific H2B.Z, establishes a H2A.Z/H2B.Z double-variant nucleosome subtype enriched at AT-rich promoter regions and correlates with open chromatin and active gene transcription [35].

Within the confined space of the nucleus, chromosomes are tightly packed into a three-dimensional structure. This three-dimensional architecture allows interaction between otherwise distant chromatin regions possessing regulatory function and facilitates contacts with other nuclear sub-compartments such as the nucleolus and the nuclear envelope [36]. Until recently, knowledge of the chromosome architecture and chromatin interactions in *Plasmodium* was mostly restricted to single genomic loci based on fluorescence *in situ* hybridization experiments [37]. However, recent advances in deep-sequencing technologies [38] have for the first time enabled the genome-wide profiling of chromosome interactions at kilobase resolution in *Plasmodium* [37, 39]. In contrast to other eukaryotic organisms, the Pf nucleus appears to lack clearly defined chromosome territories and chromatin interactions are mainly restricted to intra-chromosomal contacts showing a clear distance-related dependency [37, 39]. Inter-chromosomal interactions are mostly absent in Pf and restricted to centromeres, telomeres, ribosomal DNA (rDNA) loci, and internal as well as subtelomeric-localized *var* genes (further discussed in the next section). This observed clustering appears to coincide with transcriptional activity of each cluster. Interestingly, using three-dimensional chromatin modeling, the highly transcribed rDNA genes were proposed to be localized to the nuclear periphery, which was previously mainly associated with transcriptionally silenced heterochromatin [40], indicative of perinuclear transcriptionally active compartments [37].

Transcription itself is initiated through binding of the transcriptional machinery to promoter regions in the nucleus, resulting in the synthesis of pre-mRNA molecules, which, following extensive processing and nuclear export, leads to the accumulation of mature mRNAs in the parasite cytosol [41]. A recent study found evidence for stage-specific transcription initiation from distinct TSSs of otherwise identical transcriptional units, giving rise to developmentally regulated mRNA isoforms [42]. While most canonical eukaryotic transcription factors are absent from the *Plasmodium* genome [2], the ApiAP2 family of DNA-binding proteins comprises by far the largest group of transcription factors in malaria parasites [43]. A collection of ApiAP2 proteins is expressed throughout all stages of the IDC [44], while other ApiAP2 proteins are expressed outside the IDC [45–47]. ApiAP2s appear to be among the main drivers of developmental progress throughout most *Plasmodium* lifecycle stages and their disruption abolishes or greatly reduces parasite development [45, 46]. They bind in a sequence-specific fashion to motifs generally distributed upstream of open reading frames (ORFs) and individual AP2s may have widespread influence; PfAP2-O has been shown to bind upstream of >500 genes (roughly 10 % of the parasite ORFs), potentially influencing a wide range of cellular activities [48].

Through forward genetic screens and comparative genomics, ApiAP2-G was discovered to function as a conserved master regulator of sexual commitment in Pf and Pb. ApiAP2-G binds to a conserved 6/8-mer nucleotide motif enriched upstream of gametocyte-specific genes and *ap2-g* itself, leading to an autoregulatory feedback loop [49, 50] (Fig. 3b). ApiAP2-G2, another ApiAP2 family member, acts downstream of ApiAP2-G during sexual development, functioning as a transcriptional repressor blocking expression of genes required for asexual development and influencing gametocyte sex ratios [50, 51]. During the asexual IDC, *ap2-g* displays characteristics of epigenetically silenced heterochromatin, such as H3K9me3 marks, binding to HP1 and localization to the nuclear periphery (reviewed in [52]) (Fig. 3b). However, the previously mentioned knockdowns of both PfHDA2 and HP1 resulted in increased gametocyte conversion, likely as a direct consequence of the loss of H3K9me3 marks and H3K9 hyperacetylation leading to *ap2-g* transcriptional activation [27, 29]. This opens the possibility of a bet-hedging mechanism for sexual commitment in *Plasmodium*, regulating stochastic, low-level activation of *ap2-g* sensitive to environmental stimuli, as has been shown for several blood-stage-expressed genes [52, 53]. PTMs such as lysine acetylation are not restricted to histones and a recent study has demonstrated that the “acetylome” impacts >1000 proteins and intriguingly is highly enriched in the ApiAP2 transcription factor family [54, 55], although the functional consequences of these PTMs have yet to be established.

Following their synthesis, eukaryotic mRNAs are processed and are finally translated by the ribosomal machinery. Translation has long been a focus of malaria research, not only because it represents a promising target for antimalarial drugs but also for its potential regulatory features [56]. The lack of correlation between transcript and protein level observed throughout the *Plasmodium* lifecycle has fueled researchers’ interest in post-transcriptional and translational control for decades [57]. Many features of post-transcriptional/translational control in malaria parasites are similar to the mechanisms found in other eukaryotes [41]. However, the advent of ribosome profiling [58] has enabled in-depth genome-wide analysis of the *Plasmodium* translatome. Throughout the IDC, transcription and translation are tightly coupled and only 8 % (approximately 300 transcripts) of the transcriptome was found to be translationally regulated [59]. These genes were found to be involved in merozoite egress and invasion, and while transcript level peaked during the late stages of the IDC, maximal translation was observed during the early ring stage. This observation resembles a general feature of gene expression in *Plasmodium*, whereby for a set of genes transcription and translation are uncoupled and mRNA translation occurs during a later developmental time point when compared with maximal transcriptional activity, most notably in female gametocytes [46, 60–64]. This is especially true for genes required for developmental progression and provides the parasite with the capability of rapid and timely protein synthesis without the need for preceding de novo mRNA synthesis. Recently, PfALBA1, a member of the DNA/RNA-binding Alba protein family, was postulated to act as master regulator during the Pf IDC, controlling translation of invasion-related transcripts (Fig. 3a) as well as regulating mRNA homeostasis of approximately 100 transcripts in blood-stage parasites [65]. In contrast to findings by Caro and colleagues [59], an earlier study using polysome profiling found a discrepancy between steady-state mRNA level and polysome-associated mRNAs among 30 % of genes (1280 transcripts) during the Pf IDC, indicative of translationally controlled gene expression [66]. Additionally, the results of this study, as well the findings of others, suggest upstream ORF translation and stop codon read-through in Pf [67–69], but the genome-wide extent of such mechanisms in *Plasmodium* spp. remains controversial [59]. Hence, expansion of these studies to other parasite life stages, such as the gametocyte, where translational control is firmly established, would surely give further insights into the extent of translation regulation in *Plasmodium*.

In addition to canonical protein-coding mRNAs, a vast number of genes encoding different ncRNAs have been identified within the *Plasmodium* genome in recent years, which are believed to exert a variety of regulatory functions (reviewed in [70]). Circular RNAs (circRNAs) are among the newest members of the still expanding catalogue of existing ncRNAs in *Plasmodium* [17]. Host microRNAs (miRNAs) have been shown to regulate parasite translation [71], and circRNAs therefore might act as sponge for host miRNAs, a mechanism described in other organisms [72]. Recent studies have especially increased our knowledge of the role of ncRNAs in *var* gene regulation (discussed in the next section) but, nevertheless, the biological role of the vast majority of these ncRNA species remains unclear.

### Immune evasion

In their attempts to occupy a diverse range of host environments, protozoan parasites of the *Plasmodium* genus have evolved a plethora of molecular mechanisms to evade the host adaptive immune response. The host immune response to *Plasmodium* infection is dependent upon both host and parasite genomics and the developmental stage and phenotype of the invading parasite [73–75]. In the best-studied example in *Plasmodium*, virulence of Pf is attributed largely to monoallelic expression of just one of approximately 60 *var* genes that encode variant copies of the surface antigen, Pf erythrocyte membrane protein 1 (PfEMP1). The ability to switch expression from one *var* gene to another enables the invading parasite to alternate between phenotypes of variable cytoadherent and immunogenic properties [76–78]. PfEMP1 proteins are expressed at parasite-induced knobs on the infected erythrocyte surface, which are electron-dense features comprising many parasite proteins anchored to the erythrocyte cytoskeleton. Failure to present PfEMP1 in such knob structures greatly reduces the ability of the infected erythrocyte to bind to its specific host receptor [79].

Pf *var* gene regulation is complex and includes mechanisms of gene regulation such as chromosomal organization and subnuclear compartmentalization [80, 81], endogenous *var* gene clustering and *var* promoter–intron pairing [82, 83], transcriptional gene silencing via exoribonuclease-mediated RNA degradation [84], histone variant exchange at *var* promoters [85, 86], the effect of *trans* antisense long non-coding RNAs (lncRNAs) [87], and the presence or absence of histone modifications and their associated histone-modifying enzymes [27, 29, 40, 87–92] (Fig. 3c). Interest in delineating these mechanisms has continued, and even grown, as more research in the post-genomic area has highlighted the important differential role of the 5′ upstream promoter families into which the *var* genes can be subdivided into five classes (*upsA* to *upsE*), which correlate closely with the severity of malaria infection in the human host [93–98]. Pf *var* gene promoters are also essential components of the gene silencing mechanism and monoallelic expression. The *upsC var* promoter in particular is necessary to maintain chromosome-internal *var* genes in their silenced state and recently has been proposed to do so through the interaction of *cis*-acting MEE2-like sequence motifs and MEE2-interacting factors to reinforce *var* gene transcriptional repression [75, 83].

Monoallelic *var* gene transcription is also associated with the presence of H3K9me3 repressive marks at silent *var* gene loci (Fig. 3c). This histone modification is predicted but not proven to be imposed by the HKMT PfSET3 and is associated with perinuclear repressive centers and the binding of PfHP1, stimulating heterochromatin formation [40, 89, 90, 92]. Conditional disruption of one of these essential proteins, HP1, disrupts singular *var* gene expression and dysregulates antigenic variation [29]. In addition, conditional knockdown of PfHDA2 has been shown to result in a dramatic loss of monoallelic *var* gene expression [27]. This implicated PfHDA2 as an upstream regulator of HP1 binding as it facilitates the establishment of the H3K9me3 mark. The indispensable role of the dynamic histone lysine methylation of *Plasmodium* chromatin by histone lysine demethylases (HKDMs) and HKMTs in controlling the transcription of nearly all *var* genes has also been demonstrated. Knockout of the Pf *hkmt* gene encoding SET2/SETvs (vs, variant-silencing) resulted in reduced presence of the repressive H3K36me3 mark at the TSSs and intronic promoters of all *var* gene subtypes (Fig. 3c). Loss of this SETvs-dependent histone modification resulted in the loss of monoallelic *var* gene expression and expression of the entire *var* repertoire [98]. Furthermore, SETvs can directly interact with the C-terminal domain of RNA polymerase II, with SETvs disruption resulting in a loss of binding to RNA polymerase II and *var* gene switching [99].

Pf *upsA*-type *var* gene expression is also regulated by PfRNaseII, a chromatin-associated exoribonuclease. An inverse relationship exists between transcript levels of PfRNaseII and *upsA*-type *var* genes, with an increase in the latter corresponding to incidences of severe malaria in infected patients [84]. PfRNaseII is proposed to control *upsA*-type *var* gene transcription by marking TSSs and intronic promoter regions, degrading potential full-length transcripts to produce short-lived cryptic RNA molecules that are then further degraded by the exosome immediately upon expression (Fig. 3c). Disruption of the *pfrnaseII* gene resulted in loss of this degradation and the generation of full-length *upsA var* gene transcripts and intron-derived antisense lncRNA. These data illustrate the relationship between PfRNaseII and the control of monoallelic *var* gene transcription and suggest a correlation between lncRNA and *var* gene activation in Pf [84]. The role of lncRNAs in Pf *var* gene activation was again investigated in a study by Amit-Avraham and colleagues [87], which demonstrated dose-dependent transcriptional activation of *var* genes by overexpression of their individual antisense lncRNA transcripts. Disruption of antisense lncRNA expression by peptic nucleic acids resulted in downregulation of active *var* gene transcripts and induced *var* gene switching. The exact mechanism by which antisense lncRNAs act to promote the active transcription of a *var* gene is unknown. It has been postulated that antisense *var* transcripts may recruit chromatin-modifying enzymes that in turn would affect gene accessibility for the Pf transcriptional machinery. Antisense *var* gene lncRNAs would also contain a complementary sequence to *var* gene intronic insulator-like pairing elements that bind specific nuclear-binding proteins, therefore blocking the silencing activity of pairing elements by hybridization [87, 100].

The *Plasmodium* helical interspersed subtelomeric protein (PHIST) family of genes, which is unique to Pf, has also been implicated in the regulation of immune evasion as a result of its ability to bind to the intracellular acidic terminal segment of PfEMP1. Conditional knockdown of the essential PHIST protein PfE1605w reduced the capability of the infected host erythrocyte to adhere to the CD36 endothelial receptor, an important virulence feature of Pf. This study highlighted the importance not only of *var* genes and their controlled expression but also of other genes that are associated with anchoring PfEMP1 to the erythrocyte surface and creating the *Plasmodium* cytoadherence complex [101].

The list of regulatory mechanisms underlying *var* gene monoallelic expression is vast and much more may still be discovered in this area. However, immune evasion in the *Plasmodium* genus is not confined to Pf or *var* gene regulation. Indeed, *var* gene expression is exclusive to Pf, with much still to be gleaned in the areas of immune evasion in human malaria parasites such as *P. vivax*, *P. knowlesi*, *Plasmodium ovale* and *Plasmodium malariae* [13, 102–105]. In addition, PfEMP1 is just one of a number of variant surface antigens (VSAs) known to be expressed at the host erythrocyte surface upon infection with Pf, although it is the best characterized. Pf-infected erythrocytes also express VSAs of the multi-copy gene families of the proteins repetitive interspersed family (RIFIN), subtelomeric variable open reading frame (STEVOR), and Pf Maurer’s cleft 2 transmembrane (PfMC-2TM) [106]. The roles of these protein families in antigenic variation and pathology are generally poorly defined but are being elucidated; for example, RIFINs are implicated in the severity of Pf malaria in African children with blood group A. This tendency toward increased malaria pathogenicity is a result of their expression at the surface of infected host erythrocytes, from which they bind uninfected erythrocytes (preferentially, blood group A) to form rosette structures and mediate binding to the host microvasculature [107]. Thus, the combined roles of HP1 and HDA2 in regulating single *var* gene expression and the transcriptional regulator ApiAP2-G suggests that both processes share epigenetic regulatory mechanisms and that *Plasmodium* immune evasion and transmission to new hosts are inextricably linked [27, 29].

Immune evasion is not restricted to blood-stage *Plasmodium*; when the parasite passes through the mosquito it must also combat a sophisticated innate immune system that is very effective in reducing the parasite burden experienced by the vector. A forward genetic screen and WGS was used to identify the key parasite factor, the surface protein PfS47 (found on the surface of the ookinete as it penetrates the mosquito midgut), that appears to interact with and suppress the vector innate immune system [108]. PfS47 is thought to suppress signaling through the c-Jun N-terminal kinase (JNK) pathway that is critical to an effective immune response [109]. WGS demonstrated that PfS47 has a distinct population structure linked to global distribution. PfS47 is rapidly evolving and selected to achieve JNK suppression in diverse mosquito species, which becomes a key step in the adaptation of Pf to transmission in different vectors, thereby contributing to its broad global distribution [110].

### Artemisinin resistance

The goals of MalariaGEN characterize a new approach to understanding parasite population biology. Through the generation and, these days more critically, the management and analysis of the colossal datasets that result from WGS of large numbers of samples, a well-organized study can draw meaningful conclusions. This was applied to perhaps the most serious threat to malaria control that has emerged in recent years—resistance to ART. Using these datasets in meta-analyses with clinical data describing the individual WGS-sequenced samples and outcomes of ART treatment allowed a path to be charted that associated SNPs with treatment features (such as delayed clearance) [111] and identified candidate genes [112]: in both studies a region of chromosome 13 was implicated (Fig. 3d). The precise gene encoding the protein KELCH13 was identified by a combination of “old-fashioned” selection of drug-resistant parasites in the laboratory followed by WGS and comparative genomics of the sensitive parental parasites and the progeny, as well as WGS of ART-resistant field isolates [113, 114]. The role of the *kelch13* mutations in ART resistance was proven by direct genome engineering of *kelch13* to generate resistant parasites [115, 116]. *kelch13* SNPs have been used to map the alarmingly rapid spread of resistance throughout Southeast Asia [116] and it is clear that there is already significant but distinct *kelch13* heterogeneity in African Pf strains, although there is no evidence of ART resistance [117–121]. However, in-depth analysis of Southeast Asian ART-resistant parasite genomes [122] revealed that a complex array of background mutations (Fig. 3d) in a variety of genes (encoding ferredoxin (FD), apicoplast ribosomal protein S10 (ARPS10), multidrug resistance protein 2 (MDR2), and Pf chloroquine resistance transporter (CRT)) that are not yet described in African parasites would explain why ART resistance is not (yet) a threat to the use of ART in that continent [121].

A further puzzle was the large number of independent SNPs that seemed capable of mediating ART resistance—typically drug resistance is generated by one or a small number of SNPs focused on either altering the target binding site for the drug or preventing drug access to a binding site buried in the target structure. KELCH proteins are propeller proteins with an iterated structural motif that serves as a platform for the assembly of multi-protein complexes. In addition, KELCH13 has a BTB/POZ domain that might be involved in homodimerization, E3 ubiquitin ligase binding, and transcriptional repression (reviewed in [123]). It has been suggested that ART-resistance-associated *kelch13* SNPs might cause a degree of reduced binding of Pf phosphatidylinositol-3-kinase (PI3K), which in turn results in its reduced ubiquitination and consequent degradation of PI3K (Fig. 3d). Elevated levels of PI3K generate increased amounts of its lipid product phosphatidylinositol-3-phosphate (PI3P), which then changes the physiological state of the parasite cell through signaling in as yet unknown pathways [124] but through a mechanism predicated on the proposed abundance of PI3P in the lumen of the endoplasmic reticulum and its proposed role in protein export beyond the parasite vacuole within the host cell [125]. However, aspects of this view have been challenged [126] and further studies are clearly required to resolve the possible role of PI3K signaling in ART resistance. It will be of interest to see if PI3K signaling impacts upon the unfolded protein response implicated in ART resistance using population transcriptomics [127]. The WGS data and two proteomics studies [128, 129] that demonstrate the wide variety of proteins from different cellular compartments of the target parasite that interact with activated ART together suggest that ART resistance is a pleiotropic phenomenon [123]. Therefore, other interrogations, such as metabolomics (see next section), might also be needed to gain functional insights into ART’s mode of action.

## Translational implications for malaria control

### Antimalarials

WGS has been instrumental in identifying the cellular target of novel Pf antimalarials as part of the drug discovery pipeline and in following the in vitro selection of resistant parasite lines and validation of observed genomic changes by reverse genetics as described for ART above. This approach has proved highly successful for spiralindolines [130], resulting in the identification of the target of NITD609 (also known as KAE609 or cipargamin) as the P-type ATPase PfATPase4. Furthermore, the translation elongation factor eEF2 has been identified as the target of the 2,6-disubstituted quinoline-4-carboxamide scaffold derivative DDD107498 [131]. WGS is not the only post-genome approach that is useful for determining modes of drug action; metabolomics has a similar potential for analyzing the metabolic changes produced in response to drug exposure and has been utilized in antibiotic [132] and anti-protozoan drug [133] investigations. A metabolomics-based approach also has the advantages that parasite lines resistant to the drug need not be generated and that the activity of pleiotropically acting drugs (such as ART) are directly observed rather than imputed from genomes of resistant parasites.

### Vaccines

Post-genomic approaches have also identified promising new Pf vaccine candidates. For example, Pf reticulocyte-binding protein homologue 5 (RH5) binds to the human red cell surface receptor protein basigin, an interaction that is essential for erythrocyte invasion by Pf [134]. Recent WGS studies have shown that both the host and parasite proteins are highly conserved, that antibodies to RH5 block merozoite invasion of erythrocytes [135, 136], and that basigin itself is druggable by recombinant antibodies [137]. Although RH5–basigin interaction offers great promise, the challenges for vaccine development remain considerable and many promising candidates have fallen or will fall by the wayside due to an inability to formulate them to deliver effective vaccination, massive candidate gene sequence variability, and functional non-essentiality of the candidate. WGS will help identify non- or minimally variant candidates and should prove useful in monitoring the effect of vaccination and the analysis of “breakthrough” parasites (those developing in vaccinated individuals), as described in the next section. Effective subunit vaccines will be an invaluable additional approach to vaccination, supplementing other approaches such as the use of the promising but technologically challenging attenuated whole parasite, for example, sporozoite vaccine [138].

### Surveillance

The identification of genome signatures of resistance through WGS in the laboratory and increasingly through large-scale genomic epidemiology provides a powerful tool to monitor the emergence of resistance in *Plasmodium* populations under selective pressure due to administration of both drugs and vaccines. In the case of drugs whose targets have been identified in the laboratory, specific, simple PCR-based assays can be devised. WGS of field parasites under drug pressure is still desirable, however, as alternative resistance mechanisms might emerge that would be missed by targeted assays and, with sufficient depth of sampling, new signatures of resistance would be identifiable from the sequence data. Similar surveillance of parasites that emerge post-vaccination might also be informative. An important analysis of the clinical trial of the RTS,S/AS01 malaria vaccine compared the strain-specific sequence of the gene encoding the circumsporozoite (CS) protein that comprised the vaccine with the CS gene sequences of strains in the infections actually encountered by immunized individuals (between 5 and 17 months of age) [139]. This study demonstrated that homologous protection was greater than protection against heterologous strains and that a cause of failure to protect was simply that the CS protein carried by the infecting parasites did not match that of the vaccine and so perhaps a protective effect was less likely [139]. Therefore, WGS has the power to guide vaccine design based upon the outcomes of trials.

### Gene editing

A new era of genetic engineering has dawned with the discovery and development of the bacterial guide RNA template-targeted clustered regularly interspaced short palindromic repeats (CRISPR)-Cas9 recombinases as tools for the accurate editing of genomes. The technology has been successfully adapted to many species, including *Plasmodium* [140], *Anopheles* [141, 142], and humans (discussed in [143]). Currently, applications of CRISPR-Cas9 to *Plasmodium* manipulation are restricted to reverse genetic investigations of gene function. However, with the concepts of whole (pre-erythrocytic) parasite vaccines [144, 145], CRISPR-Cas9 offers an obvious route towards the generation of an immunogenic, non-pathogenic parasite that might be suitably safe to administer to humans as a vaccination strategy. Clearly, the engineering of human genomes at any stage of gestation is fraught with ethical considerations [146] and it is inconceivable that this will be applied to the improvement of human resistance to malaria in the foreseeable future. Conversely, although subject to similar ethical and ecological debate, significant conceptual advances towards the generation of CRISPR-Cas9-engineered *Anopheles* mosquitoes have been quickly achieved. Through harnessing the concept of gene drive, two independent teams have reported the generation of either engineered *Anopheles stephensi* (a major Indian vector of malaria) that is resistant to malaria [141] or sterile female Ag [142]. Again, owing to ecological considerations, it is unlikely that such engineered mosquitoes, although clearly feasible, will be released into the wild any time soon [147].

## Conclusions and future directions

Despite the progress summarized here, the fundamental requirements of malaria research in any era remain the same; namely, new drugs to replace those that become ineffective, vaccines that work, and the means to administer them effectively. Genomics, post-genomic technologies, and associated computing developments have revolutionized investigations into the biology of the malaria parasite and the search for therapeutics or intervention measures. Significant progress has been made on many fronts, including candidate drug and vaccine discovery, parasite drug resistance mechanisms, host–parasite–vector interactions and parasite biology, and mechanisms of human resistance to malaria. Also, new concepts of combatting malaria via engineered mosquito populations have been introduced through the advent of novel genome-editing approaches such as CRISPR-Cas9.

We can anticipate that WGS will continue to improve in terms of both cost and quality, making sequencing of every desirable Pf isolate feasible. This would enable more detailed studies of population structure and dynamics, allowing the tracking of gene flow and genotype success that might even resolve at the village level and, further, potentially almost in real time. However, this will only happen if data storage, access, and computing technologies keep pace. Where Pf WGS studies have gone *P. vivax* research will follow and recent studies have revealed signatures of drug selection superimposed upon a far more complex (global, regional, and even within a single infection) population structure than Pf [148, 149]. Single-cell RNA sequencing will significantly improve our understanding of antigenic variation and variant and sex-specific gene expression.

More immediately, an important need is for surveillance, particularly in Africa, to look for *kelch13* mutations and genotypes associated with ART resistance and a pan-African network is in place to monitor for this and collect samples [150]. Genomics will continue to be used in novel ways as well, for example, in studies of the outcomes of human interventions such as drug treatment and vaccination.

New fields of endeavor are also emerging that will certainly prove fruitful in the years to come. Lipidomics is a nascent discipline that will no doubt reveal insights into membrane composition and organization [151] and might also open avenues to therapy. PTMs such as palmitoylation give proteins the means to conditionally interact with membranes and *Plasmodium* makes extensive use of protein palmitoylation that should influence a range of important parasite biological activities, such as cytoadherence and drug resistance [152].

Although the power of genomics approaches is quite clear, direct biological investigations are frequently required to confirm or refute the findings that genomics might imply. The numerous examples given here indicate that although genomic analyses often generate associations and degrees of confidence about their conclusions, unequivocal confirmation is provided by genetic engineering (of parasites and their vectors at least). Genetic screens are powerful, often unbiased approaches to discover gene function. The recent development of the PlasmoGEM resource coupled with high-efficiency transfection and barcoded vectors permits genome-scale reverse genetics screens to be deployed that will undoubtedly reveal information about parasite-specific genes and *Plasmodium* biology [153]. Finally, many of the genes encoded by parasite, host, and vector genomes have unknown functions, the details of which are slowly emerging as technologies and assays improve. The staggering complexity of organismal biology and the interactions between parasite, host, and vector will continue to amaze but equally will offer hope for new and improved therapies.

## Abbreviations

(l)ncRNA: (Long) Non-coding RNA
Ag: *Anopheles Gambiae*
AID: Auxin-inducible Degron
ART: Artemisinin
circRNA: Circular RNAs
cKD: Conditional Knockdown
CRISPR: Clustered Regularly Interspaced Short Palindromic Repeats
DD: Destabilization Domain
G6PD: Glucose-6-phosphate Dehydrogenase
GWAS: Genome-wide Association Study
IDC: Intra-erythrocytic Development Cycle
K13: Kelch13
MalariaGEN: Malaria Genomic Epidemiology Network
miRNA: MicroRNA
NGS: Next-generation Sequencing
ORF: Open Reading Frame
Pb: Plasmodium Berghei
Pf: Plasmodium Falciparum
PHIST: Plasmodium Helical Interspersed Subtelomeric Protein Family
PI3K: Phosphatidylinositol-3-kinase
PI3P: Phosphatidylinositol-3-phosphate
PTM: Post-translational Modification
RBC: Red Blood Cell
rDNA: Ribosomal DNA
RIFIN: Repetitive Interspersed Family
SNP: Single-nucleotide Polymorphism
TF: Transcription Factor
TSS: Transcription Start Site
UPR: Unfolded Protein Response
VSAs: Variant Surface Antigens
WGS: Whole-genome Sequencing
ZFN: Zinc Finger Nuclease

## References

[1] World Health Organization. World Malaria Report 2015. 2015. http://apps.who.int/iris/bitstream/10665/200018/1/9789241565158_eng.pdf?ua=1. Accessed 24 June 2016.

[2] Gardner MJ, Hall N, Fung E, White O, Berriman M, Hyman RW (2002). Genome sequence of the human malaria parasite *Plasmodium falciparum*. Nature.

[3] Larremore DB, Sundararaman SA, Liu W, Proto WR, Clauset A, Loy DE (2015). Ape parasite origins of human malaria virulence genes. Nat Commun.

[4] Kwiatkowski D (2015). Malaria genomics: tracking a diverse and evolving parasite population. Int Health.

[5] Malaria Genomic Epidemiology Network (2015). A novel locus of resistance to severe malaria in a region of ancient balancing selection. Nature.

[6] Redmond SN, Eiglmeier K, Mitri C, Markianos K, Guelbeogo WM, Gneme A (2015). Association mapping by pooled sequencing identifies TOLL 11 as a protective factor against *Plasmodium falciparum* in *Anopheles gambiae*. BMC Genomics.

[7] Gurdasani D, Carstensen T, Tekola-Ayele F, Pagani L, Tachmazidou I, Hatzikotoulas K (2015). The African Genome Variation Project shapes medical genetics in Africa. Nature.

[8] Malaria Genomic Epidemiology Network (2014). Reappraisal of known malaria resistance loci in a large multicenter study. Nat Genet.

[9] Maier AG, Duraisingh MT, Reeder JC, Patel SS, Kazura JW, Zimmerman PA (2003). *Plasmodium falciparum* erythrocyte invasion through glycophorin C and selection for Gerbich negativity in human populations. Nat Med.

[10] Fontaine MC, Pease JB, Steele A, Waterhouse RM, Neafsey DE, Sharakhov IV (2015). Extensive introgression in a malaria vector species complex revealed by phylogenomics. Science.

[11] Neafsey DE, Waterhouse RM, Abai MR, Aganezov SS, Alekseyev MA, Allen JE (2015). Highly evolvable malaria vectors: the genomes of 16 *Anopheles* mosquitoes. Science.

[12] Carlton JM, Adams JH, Silva JC, Bidwell SL, Lorenzi H, Caler E (2008). Comparative genomics of the neglected human malaria parasite *Plasmodium vivax*. Nature.

[13] Pain A, Böhme U, Berry AE, Mungall K, Finn RD, Jackson AP (2008). The genome of the simian and human malaria parasite *Plasmodium knowlesi*. Nature.

[14] Keeling PJ, Rayner JC (2015). The origins of malaria: there are more things in heaven and earth. Parasitology.

[15] Liu W, Sundararaman SA, Loy DE, Learn GH, Li Y, Plenderleith LJ (2016). Multigenomic delineation of *Plasmodium* species of the *Laverania* subgenus infecting wild-living chimpanzees and gorillas. Genome Biol Evol.

[16] Mourier T, Carret C, Kyes S, Christodoulou Z, Gardner PP, Jeffares DC (2008). Genome-wide discovery and verification of novel structured RNAs in *Plasmodium falciparum*. Genome Res.

[17] Broadbent KM, Broadbent JC, Ribacke U, Wirth D, Rinn JL, Sabeti PC (2015). Strand-specific RNA sequencing in *Plasmodium falciparum* malaria identifies developmentally regulated long non-coding RNA and circular RNA. BMC Genomics.

[18] Siegel TN, Hon CC, Zhang Q, Lopez-Rubio JJ, Scheidig-Benatar C, Martins RM (2014). Strand-specific RNA-Seq reveals widespread and developmentally regulated transcription of natural antisense transcripts in *Plasmodium falciparum*. BMC Genomics.

[19] Manske M, Miotto O, Campino S, Auburn S, Almagro-Garcia J, Maslen G (2012). Analysis of *Plasmodium falciparum* diversity in natural infections by deep sequencing. Nature.

[20] Bonasio R, Tu S, Reinberg D (2010). Molecular signals of epigenetic states. Science.

[21] Cui L, Miao J (2010). Chromatin-mediated epigenetic regulation in the malaria parasite *Plasmodium falciparum*. Eukaryot Cell.

[22] Saraf A, Cervantes S, Bunnik EM, Ponts N, Sardiu ME, Chung DD, et al. Dynamic and combinatorial landscape of histone modifications during the intraerythrocytic developmental cycle of the malaria parasite. J Proteome Res. 2016; doi:10.1021/acs.jproteome.6b00366. 10.1021/acs.jproteome.6b00366 PMC5905347 27291344

[23] Volz J, Carvalho TG, Ralph SA, Gilson P, Thompson J, Tonkin CJ (2010). Potential epigenetic regulatory proteins localise to distinct nuclear sub-compartments in *Plasmodium falciparum*. Int J Parasitol.

[24] Doerig C, Rayner JC, Scherf A, Tobin AB (2015). Post-translational protein modifications in malaria parasites. Nat Rev Microbiol.

[25] Chen PB, Ding S, Zanghì G, Soulard V, Dimaggio PA, Fuchter MJ (2016). *Plasmodium falciparum Pf*SET7: enzymatic characterization and cellular localization of a novel protein methyltransferase in sporozoite, liver and erythrocytic stage parasites. Sci Rep.

[26] Biamonte MA, Wanner J, Le Roch KG (2013). Recent advances in malaria drug discovery. Bioorg Med Chem Lett.

[27] Coleman BI, Skillman KM, Jiang RH, Childs LM, Altenhofen LM, Ganter M (2014). A *Plasmodium falciparum* histone deacetylase regulates antigenic variation and gametocyte conversion. Cell Host Microbe.

[28] Bártfai R, Hoeijmakers WA, Salcedo-Amaya AM, Smits AH, Janssen-Megens E, Kaan A (2010). H2A.Z demarcates intergenic regions of the *plasmodium falciparum* epigenome that are dynamically marked by H3K9ac and H3K4me3. PLoS Pathog.

[29] Brancucci NM, Bertschi NL, Zhu L, Niederwieser I, Chin WH, Wampfler R (2014). Heterochromatin protein 1 secures survival and transmission of malaria parasites. Cell Host Microbe.

[30] Lomberk G, Wallrath L, Urrutia R (2006). The heterochromatin protein 1 family. Genome Biol.

[31] Josling GA, Petter M, Oehring SC, Gupta AP, Dietz O, Wilson DW (2015). A *Plasmodium falciparum* bromodomain protein regulates invasion gene expression. Cell Host Microbe.

[32] Bunnik EM, Polishko A, Prudhomme J, Ponts N, Gill SS, Lonardi S (2014). DNA-encoded nucleosome occupancy is associated with transcription levels in the human malaria parasite *Plasmodium falciparum*. BMC Genomics.

[33] Ponts N, Harris EY, Prudhomme J, Wick I, Eckhardt-Ludka C, Hicks GR (2010). Nucleosome landscape and control of transcription in the human malaria parasite. Genome Res.

[34] Kensche PR, Hoeijmakers WAM, Toenhake CG, Bras M, Chappell L, Berriman M, et al. The nucleosome landscape of *Plasmodium falciparum* reveals chromatin architecture and dynamics of regulatory sequences. Nucleic Acids Res. 2015. doi:10.1093/nar/gkv1214. 10.1093/nar/gkv1214 PMC4797266 26578577

[35] Hoeijmakers WA, Salcedo-Amaya AM, Smits AH, Françoijs KJ, Treeck M, Gilberger TW (2013). H2A.Z/H2B.Z double-variant nucleosomes inhabit the AT-rich promoter regions of the *Plasmodium falciparum* genome. Mol Microbiol.

[36] Pombo A, Dillon N (2015). Three-dimensional genome architecture: players and mechanisms. Nat Rev Mol Cell Biol.

[37] Ay F, Bunnik EM, Varoquaux N, Bol SM, Prudhomme J, Vert JP (2014). Three-dimensional modeling of the *P. falciparum* genome during the erythrocytic cycle reveals a strong connection between genome architecture and gene expression. Genome Res.

[38] de Wit E, de Laat W (2012). A decade of 3C technologies: insight into nuclear organization. Genes Dev.

[39] Lemieux JE, Kyes SA, Otto TD, Feller AI, Eastman RT, Pinches RA (2013). Genome-wide profiling of chromosome interactions in *Plasmodium falciparum* characterizes nuclear architecture and reconfigurations associated with antigenic variation. Mol Microbiol.

[40] Lopez-Rubio JJ, Mancio-Silva L, Scherf A (2009). Genome-wide analysis of heterochromatin associates clonally variant gene regulation with perinuclear repressive centers in malaria parasites. Cell Host Microbe.

[41] Vembar SS, Droll D, Scherf A. Translational regulation in blood stages of the malaria parasite *Plasmodium* spp.: systems-wide studies pave the way. Wiley Interdiscip Rev RNA. 2016. doi:10.1002/wrna.1365. 10.1002/wrna.1365 PMC5111744 27230797

[42] Adjalley SH, Chabbert CD, Klaus B, Pelechano V, Steinmetz LM (2016). Landscape and dynamics of transcription initiation in the malaria parasite *Plasmodium falciparum*. Cell Rep.

[43] Balaji S, Babu MM, Iyer LM, Aravind L (2005). Discovery of the principal specific transcription factors of Apicomplexa and their implication for the evolution of the AP2-integrase DNA binding domains. Nucleic Acids Res.

[44] Painter HJ, Campbell TL, Llinás M (2011). The Apicomplexan AP2 family: integral factors regulating *Plasmodium* development. Mol Biochem Parasitol.

[45] Iwanaga S, Kaneko I, Kato T, Yuda M (2012). Identification of an AP2-family protein that is critical for malaria liver stage development. PLoS One.

[46] Yuda M, Iwanaga S, Shigenobu S, Mair GR, Janse CJ, Waters AP (2009). Identification of a transcription factor in the mosquito-invasive stage of malaria parasites. Mol Microbiol.

[47] Yuda M, Iwanaga S, Shigenobu S, Kato T, Kaneko I (2010). Transcription factor AP2-Sp and its target genes in malarial sporozoites. Mol Microbiol.

[48] Kaneko I, Iwanaga S, Kato T, Kobayashi I, Yuda M (2015). Genome-wide identification of the target genes of AP2-O, a *Plasmodium* AP2-family transcription factor. PLoS Pathog.

[49] Kafsack BF, Rovira-Graells N, Clark TG, Bancells C, Crowley VM, Campino SG (2014). A transcriptional switch underlies commitment to sexual development in malaria parasites. Nature.

[50] Sinha A, Hughes KR, Modrzynska KK, Otto TD, Pfander C, Dickens NJ (2014). A cascade of DNA-binding proteins for sexual commitment and development in *Plasmodium*. Nature.

[51] Yuda M, Iwanaga S, Kaneko I, Kato T (2015). Global transcriptional repression: an initial and essential step for *Plasmodium sexual* development. Proc Natl Acad Sci U S A.

[52] Waters AP (2016). Epigenetic roulette in blood stream *Plasmodium*: gambling on sex. PLoS Path.

[53] Rovira-Graells N, Gupta AP, Planet E, Crowley VM, Mok S, Ribas de Pouplana L (2012). Transcriptional variation in the malaria parasite *Plasmodium falciparum*. Genome Res.

[54] Miao J, Lawrence M, Jeffers V, Zhao F, Parker D, Ge Y (2013). Extensive lysine acetylation occurs in evolutionarily conserved metabolic pathways and parasite-specific functions during *Plasmodium falciparum* intraerythrocytic development. Mol Microbiol.

[55] Cobbold SA, Santos JM, Ochoa A, Perlman DH, Llinás M (2016). Proteome-wide analysis reveals widespread lysine acetylation of major protein complexes in the malaria parasite. Sci Rep.

[56] Jackson KE, Habib S, Frugier M, Hoen R, Khan S, Pham JS (2011). Protein translation in *Plasmodium* parasites. Trends Parasitol.

[57] Le Roch KG, Zhou Y, Blair PL, Grainger M, Moch JK, Haynes JD (2003). Discovery of gene function by expression profiling of the malaria parasite life cycle. Science.

[58] Ingolia NT, Ghaemmaghami S, Newman JR, Weissman JS (2009). Genome-wide analysis in vivo of translation with nucleotide resolution using ribosome profiling. Science.

[59] Caro F, Ahyong V, Betegon M, DeRisi JL (2014). Genome-wide regulatory dynamics of translation in the *Plasmodium falciparum* asexual blood stages. Elife.

[60] Mair GR, Braks JA, Garver LS, Wiegant JC, Hall N, Dirks RW (2006). Regulation of sexual development of *Plasmodium* by translational repression. Science.

[61] Silva PA, Guerreiro A, Santos JM, Braks JA, Janse CJ, Mair GR (2016). Translational control of UIS4 protein of the host-parasite interface is mediated by the RNA binding protein Puf2 in *Plasmodium berghei* sporozoites. PLoS One.

[62] Gomes-Santos CSS, Brak JAM, Prudêncio M, Carret CK, Gomes ARB, Pain A (2011). *Plasmodium* sporozoites in the salivary gland are latent liver stage forms regulated by the RNA binding protein pumilio. PloS Path.

[63] Lasonder E, Rijpma SR, Van Schaijk BC, Hoeijmakers WA, Kensche PR, Gresnigt MS, et al. Integrated transcriptomic and proteomic analyses of *P. falciparum* gametocytes: molecular insight into sex-specific processes and translational repression. Nucleic Acids Res. 2016. doi:10.1093/nar/gkw536. 10.1093/nar/gkw536 PMC5291273 27298255

[64] Guerreiro A, Deligianni E, Santos JM, Silva PA, Louis C, Pain A (2014). Genome-wide RIP-Chip analysis of translational repressor-bound mRNAs in the *Plasmodium* gametocyte. Genome Biol.

[65] Vembar SS, Macpherson CR, Sismeiro O, Coppée JY, Scherf A (2015). The *Pf*Alba1 RNA-binding protein is an important regulator of translational timing in *Plasmodium falciparum* blood stages. Genome Biol.

[66] Bunnik EM, Chung DW, Hamilton M, Ponts N, Saraf A, Prudhomme J (2013). Polysome profiling reveals translational control of gene expression in the human malaria parasite *Plasmodium falciparum*. Genome Biol.

[67] Brancucci NM, Witmer K, Schmid C, Voss TS (2014). A *var* gene upstream element controls protein synthesis at the level of translation initiation in *Plasmodium falciparum*. PLoS One.

[68] Kumar M, Srinivas V, Patankar S (2015). Upstream AUGs and upstream ORFs can regulate the downstream ORF in *Plasmodium falciparum*. Malar J.

[69] Chakrabarti K, Pearson M, Grate L, Sterne-Weiler T, Deans J, Donohue JP (2007). Structural RNAs of known and unknown function identified in malaria parasites by comparative genomics and RNA analysis. RNA.

[70] Vembar SS, Scherf A, Siegel TN (2014). Noncoding RNAs as emerging regulators of *Plasmodium falciparum* virulence gene expression. Curr Opin Microbiol.

[71] LaMonte G, Philip N, Reardon J, Lacsina JR, Majoros W, Chapman L (2012). Translocation of sickle cell erythrocyte microRNAs into *Plasmodium falciparum* inhibits parasite translation and contributes to malaria resistance. Cell Host Microbe.

[72] Chen LL (2016). The biogenesis and emerging roles of circular RNAs. Nat Rev Mol Cell Biol.

[73] Kwiatkowski DP (2005). How malaria has affected the human genome and what human genetics can teach us about malaria. Am J Hum Genet.

[74] Ferreira MU, Da Silva NM, Wunderlich G (2004). Antigenic diversity and immune evasion by malaria parasites. Clin Diagn Lab Immunol.

[75] Brancucci NM, Witmer K, Schmid CD, Flueck C, Voss TS (2012). Identification of a *cis-*acting DNA-protein interaction implicated in singular *var* gene choice in *Plasmodium falciparum*. Cell Microbiol.

[76] Smith JD, Chitnis CE, Craig AG, Roberts DJ, Hudson-Taylor DE, Peterson DS (1995). Switches in expression of *Plasmodium falciparum var* genes correlate with changes in antigenic and cytoadherent phenotypes of infected erythrocytes. Cell.

[77] Recker M, Buckee CO, Serazin A, Kyes S, Pinches R, Christodoulou Z (2011). Antigenic variation in *Plasmodium falciparum* malaria involves a highly structured switching pattern. PLoS Pathog.

[78] Almelli T, Ndam NT, Ezimegnon S, Alao MJ, Ahouansou C, Sagbo G (2014). Cytoadherence phenotype of *Plasmodium falciparum*-infected erythrocytes is associated with specific pfemp-1 expression in parasites from children with cerebral malaria. Malar J.

[79] Subramani R, Quadt K, Jeppesen AE, Hempel C, Petersen JE, Hassenkam T (2015). *Plasmodium falciparum*-infected erythrocyte knob density is linked to the *Pf*EMP1 variant expressed. MBio.

[80] Thompson JK, Rubio JP, Caruana S, Brockman A, Wickham ME, Cowman AF (1997). The chromosomal organization of the *Plasmodium falciparum var* gene family is conserved. Mol Biochem Parasitol.

[81] Freitas-Junior LH, Bottius E, Pirrit LA, Deitsch KW, Scheidig C, Guinet F (2000). Frequent ectopic recombination of virulence factor genes in telomeric chromosome clusters of *P. falciparum*. Nature.

[82] Swamy L, Amulic B, Deitsch KW (2011). *Plasmodium falciparum var* gene silencing is determined by *cis* DNA elements that form stable and heritable interactions. Eukaryot Cell.

[83] Voss TS, Healer J, Marty AJ, Duffy MF, Thompson JK, Beeson JG (2006). A *var* gene promoter controls allelic exclusion of virulence genes in *Plasmodium falciparum* malaria. Nature.

[84] Zhang Q, Siegel TN, Martins RM, Wang F, Cao J, Gao Q (2014). Exonuclease-mediated degradation of nascent RNA silences genes linked to severe malaria. Nature.

[85] Petter M, Lee CC, Byrne TJ, Boysen KE, Volz J, Ralph SA (2011). Expression of *P. falciparum var* genes involves exchange of the histone variant H2A.Z at the promoter. PLoS Pathog.

[86] Petter M, Selvarajah SA, Lee CC, Chin WH, Gupta AP, Bozdech Z (2013). H2A.Z and H2B.Z double-variant nucleosomes define intergenic regions and dynamically occupy *var* gene promoters in the malaria parasite *Plasmodium falciparum*. Mol Microbiol.

[87] Amit-Avraham I, Pozner G, Eshar S, Fastman Y, Kolevzon N, Yavin E (2015). Antisense long noncoding RNAs regulate *var* gene activation in the malaria parasite *Plasmodium falciparum*. Proc Natl Acad Sci U S A.

[88] Lopez-Rubio JJ, Gontijo AM, Nunes MC, Issar N, Hernandez Rivas R, Scherf A (2007). 5′ flanking region of *var* genes nucleate histone modification patterns linked to phenotypic inheritance of virulence traits in malaria parasites. Mol Microbiol.

[89] Flueck C, Bartfai R, Volz J, Niederwieser I, Salcedo-Amaya AM, Alako BT (2009). *Plasmodium falciparum* heterochromatin protein 1 marks genomic loci linked to phenotypic variation of exported virulence factors. PLoS Pathog.

[90] Pérez-Toledo K, Rojas-Meza AP, Mancio-Silva L, Hernández-Cuevas NA, Delgadillo DM, Vargas M (2009). *Plasmodium falciparum* heterochromatin protein 1 binds to tri-methylated histone 3 lysine 9 and is linked to mutually exclusive expression of *var* genes. Nucleic Acids Res.

[91] Duraisingh MT, Voss TS, Marty AJ, Duffy MF, Good RT, Thompson JK (2005). Heterochromatin silencing and locus repositioning linked to regulation of virulence genes in *Plasmodium falciparum*. Cell.

[92] Freitas-Junior LH, Hernandez-Rivas R, Ralph SA, Montiel-Condado D, Ruvalcaba-Salazar OK, Rojas-Meza AP (2005). Telomeric heterochromatin propagation and histone acetylation control mutually exclusive expression of antigenic variation genes in malaria parasites. Cell.

[93] Su XZ, Heatwole VM, Wertheimer SP, Guinet F, Herrfeldt JA, Peterson DS (1995). The large diverse gene family *var* encodes proteins involved in cytoadherence and antigenic variation of *Plasmodium falciparum*-infected erythrocytes. Cell.

[94] Lavstsen T, Salanti A, Jensen AT, Arnot DE, Theander TG (2003). Sub-grouping of *Plasmodium falciparum* 3D7 *var* genes based on sequence analysis of coding and non-coding regions. Malar J.

[95] Kyriacou HM, Stone GN, Challis RJ, Raza A, Lyke KE, Thera MA (2006). Differential *var* gene transcription in *Plasmodium falciparum* isolates from patients with cerebral malaria compared to hyperparasitaemia. Mol Biochem Parasitol.

[96] Kyes SA, Kraemer SM, Smith JD (2007). Antigenic variation in *Plasmodium falciparum*: gene organization and regulation of the *var* multigene family. Eukaryot Cell.

[97] Trimnell AR, Kraemer SM, Mukherjee S, Phippard DJ, Janes JH, Flamoe E (2006). Global genetic diversity and evolution of *var* genes associated with placental and severe childhood malaria. Mol Biochem Parasitol.

[98] Jiang L, Mu J, Zhang Q, Ni T, Srinivasan P, Rayavara K (2013). PfSETvs methylation of histone H3K36 represses virulence genes in *Plasmodium falciparum*. Nature.

[99] Ukaegbu UE, Kishore SP, Kwiatkowski DL, Pandarinath C, Dahan-Pasternak N, Dzikowski R (2014). Recruitment of *Pf*SET2 by RNA polymerase II to variant antigen encoding loci contributes to antigenic variation in *P. falciparum*. Plos Pathog.

[100] Avraham I, Schreier J, Dzikowski R (2012). Insulator-like pairing elements regulate silencing and mutually exclusive expression in the malaria parasite *Plasmodium falciparum*. Proc Natl Acad Sci U S A.

[101] Oberli A, Zurbrügg L, Rusch S, Brand F, Butler ME, Day JL, et al. *Plasmodium falciparum* PHIST proteins contribute to cytoadherence and anchor *Pf*EMP1 to the host cell cytoskeleton. Cell Microbiol. 2016. doi:10.1111/cmi.12583. 10.1111/cmi.12583 PMC5103180 26916885

[102] Flick K, Chen Q (2004). *var* genes, *Pf*EMP1 and the human host. Mol Biochem Parasitol.

[103] Lapp SA, Korir-Morrison C, Jiang J, Bai Y, Corredor V, Galinski MR (2013). Spleen-dependent regulation of antigenic variation in malaria parasites: *Plasmodium knowlesi* SICAvar expression profiles in splenic and asplenic hosts. PLoS One.

[104] Neafsey DE, Galinsky K, Jiang RH, Young L, Sykes SM, Saif S (2012). The malaria parasite *Plasmodium vivax* exhibits greater genetic diversity than *Plasmodium falciparum*. Nat Genet.

[105] Fernandez-Becerra C, Yamamoto MM, Vêncio RZ, Lacerda M, Rosanas-Urgell A, Del Portillo HA (2009). *Plasmodium vivax* and the importance of the subtelomeric multigene *vir* superfamily. Trends Parasitol.

[106] Frech C, Chen N (2013). Variant surface antigens of malaria parasites: functional and evolutionary insights from comparative gene family classification and analysis. BMC Genomics.

[107] Bachmann A, Scholz JA, Janssen M, Klinkert MQ, Tannich E, Bruchhaus I (2015). A comparative study of the localization and membrane topology of members of the RIFIN, STEVOR and PfMC-2TM protein families in *Plasmodium falciparum*-infected erythrocytes. Malar J.

[108] Molina-Cruz A, Garver LS, Alabaster A, Bangiolo L, Haile A, Winikor J (2013). The human malaria parasite Pfs47 gene mediates evasion of the mosquito immune system. Science.

[109] Ramphul UN, Garver LS, Molina-Cruz A, Canepa GE, Barillas-Mury C (2015). *Plasmodium falciparum* evades mosquito immunity by disrupting JNK-mediated apoptosis of invaded midgut cells. Proc Natl Acad Sci U S A.

[110] Molina-Cruz A, Canepa GE, Kamath N, Pavlovic NV, Mu J, Ramphul UN (2015). *Plasmodium* evasion of mosquito immunity and global malaria transmission: the lock-and-key theory. Proc Natl Acad Sci U S A.

[111] Takala-Harrison S, Clark TG, Jacob CG, Cummings MP, Miotto O, Dondorp AM (2013). Genetic loci associated with delayed clearance of *Plasmodium falciparum* following artemisinin treatment in Southeast Asia. Proc Natl Acad Sci U S A.

[112] Cheeseman IH, Miller BA, Nair S, Nkhoma S, Tan A, Tan JC (2012). A major genome region underlying artemisinin resistance in malaria. Science.

[113] Borrmann S, Straimer J, Mwai L, Abdi A, Rippert A, Okombo J (2013). Genome-wide screen identifies new candidate genes associated with artemisinin susceptibility in *Plasmodium falciparum* in Kenya. Sci Rep.

[114] Ariey F, Witkowski B, Amaratunga C, Beghain J, Langlois AC, Khim N (2014). A molecular marker of artemisinin-resistant *Plasmodium falciparum* malaria. Nature.

[115] Ghorbal M, Gorman M, Macpherson CR, Martins RM, Scherf A, Lopez-Rubio JJ (2014). Genome editing in the human malaria parasite *Plasmodium falciparum* using the CRISPR-Cas9 system. Nat Biotechnol.

[116] Straimer J, Gnädig NF, Witkowski B, Amaratunga C, Duru V, Ramadani AP (2015). K13-propeller mutations confer artemisinin resistance in *Plasmodium falciparum* clinical isolates. Science.

[117] Ashley EA, Dhorda M, Fairhurst RM, Amaratunga C, Lim P, Suon S (2014). Spread of artemisinin resistance in *Plasmodium falciparum* malaria. N Engl J Med.

[118] Conrad MD, Bigira V, Kapisi J, Muhindo M, Kamya MR, Havlir DV (2014). Polymorphisms in K13 and falcipain-2 associated with artemisinin resistance are not prevalent in *Plasmodium falciparum* isolated from Ugandan children. PLoS One.

[119] Torrentino-Madamet M, Fall B, Benoit N, Camara C, Amalvict R, Fall M (2014). Limited polymorphisms in *k13* gene in *Plasmodium falciparum* isolates from Dakar, Senegal in 2012–2013. Malar J.

[120] Ouattara A, Kone A, Adams M, Fofana B, Maiga AW, Hampton S (2015). Polymorphisms in the K13-propeller gene in artemisinin-susceptible *Plasmodium falciparum* parasites from Bougoula-Hameau and Bandiagara. Mali Am J Trop Med Hyg.

[121] Ménard D, Khim N, Beghain J, Adegnika AA, Shafiul-Alam M, Amodu O (2016). A worldwide map of Plasmodium falciparum K13-propeller polymorphisms. N Engl J Med.

[122] Miotto O, Amato R, Ashley EA, MacInnis B, Almagro-Garcia J, Amaratunga C (2015). Genetic architecture of artemisinin-resistant *Plasmodium falciparum*. Nat Genet.

[123] Tilley L, Straimer J, Gnädig NF, Ralph SA, Fidock DA. Artemisinin action and resistance in *Plasmodium falciparum*. Trends Parasitol. 2016. doi:10.1016/j.pt.2016.05.010. 10.1016/j.pt.2016.05.010 PMC5007624 27289273

[124] Mbengue A, Bhattacharjee S, Pandharkar T, Liu H, Estiu G, Stahelin RV (2015). A molecular mechanism of artemisinin resistance in *Plasmodium falciparum* malaria. Nature.

[125] Bhattacharjee S, Stahelin RV, Speicher KD, Speicher DW, Haldar K (2012). Endoplasmic reticulum PI(3)P lipid binding targets malaria proteins to the host cell. Cell.

[126] Boddey JA, O'Neill MT, Lopaticki S, Carvalho TG, Hodder AN, Nebl T (2016). Export of malaria proteins requires co-translational processing of the PEXEL motif independent of phosphatidylinositol-3-phosphate binding. Nat Commun.

[127] Mok S, Ashley EA, Ferreira PE, Zhu L, Lin Z, Yeo T (2015). Population transcriptomics of human malaria parasites reveals the mechanism of artemisinin resistance. Science.

[128] Wang J, Zhang CJ, Chia WN, Loh CC, Li Z, Lee YM (2015). Haem-activated promiscuous targeting of artemisinin in *Plasmodium falciparum*. Nat Commun.

[129] Ismail HM, Barton V, Phanchana M, Charoensutthivarakul S, Wong MH, Hemingway J (2016). Artemisinin activity-based probes identify multiple molecular targets within the asexual stage of the malaria parasites *Plasmodium falciparum* 3D7. Proc Natl Acad Sci U S A.

[130] Rottmann M, McNamara C, Yeung BK, Lee MC, Zou B, Russell B (2010). Spiroindolones, a potent compound class for the treatment of malaria. Science.

[131] Baragaña B, Hallyburton I, Lee MC, Norcross NR, Grimaldi R, Otto TD (2015). A novel multiple-stage antimalarial agent that inhibits protein synthesis. Nature.

[132] Vincent IM, Ehmann DE, Mills SD, Perros M, Barrett MP (2016). Untargeted metabolomics to ascertain antibiotic modes of action. Antimicrob Agents Chemother.

[133] Vincent IM, Creek DJ, Burgess K, Woods DJ, Burchmore RJ, Barrett MP (2012). Untargeted metabolomics reveals a lack of synergy between nifurtimox and eflornithine against *Trypanosoma brucei*. PLoS Negl Trop Dis.

[134] Crosnier C, Bustamante LY, Bartholdson SJ, Bei AK, Theron M, Uchikawa M (2011). Basigin is a receptor essential for erythrocyte invasion by *Plasmodium falciparum*. Nature.

[135] Douglas AD, Williams AR, Knuepfer E, Illingworth JJ, Furze JM, Crosnier C (2014). Neutralization of *Plasmodium falciparum* merozoites by antibodies against *Pf*RH5. J Immunol.

[136] Wright KE, Hjerrild KA, Bartlett J, Douglas AD, Jin J, Brown RE (2014). Structure of malaria invasion protein RH5 with erythrocyte basigin and blocking antibodies. Nature.

[137] Zenonos ZA, Dummler SK, Müller-Sienerth N, Chen J, Preiser PR, Rayner JC (2015). Basigin is a druggable target for host-oriented antimalarial interventions. J Exp Med.

[138] Ishizuka AS, Lyke KE, DeZure A, Berry AA, Richie TL, Mendoza FH (2016). Protection against malaria at 1 year and immune correlates following PfSPZ vaccination. Nat Med.

[139] Neafsey DE, Juraska M, Bedford T, Benkeser D, Valim C, Griggs A (2015). Genetic diversity and protective efficacy of the RTS, S/AS01 malaria vaccine. N Engl J Med.

[140] Wagner JC, Platt RJ, Goldfless SJ, Zhang F, Niles JC (2014). Efficient CRISPR-Cas9-mediated genome editing in *Plasmodium falciparum*. Nat Methods.

[141] Gantz VM, Jasinskiene N, Tatarenkova O, Fazekas A, Macias VM, Bier E (2015). Highly efficient Cas9-mediated gene drive for population modification of the malaria vector mosquito *Anopheles stephensi*. Proc Natl Acad Sci U S A.

[142] Hammond A, Galizi R, Kyrou K, Simoni A, Siniscalchi C, Katsanos D (2016). A CRISPR-Cas9 gene drive system targeting female reproduction in the malaria mosquito vector *Anopheles gambiae*. Nat Biotechnol.

[143] Callaway E (2016). Gene-editing research in human embryos gains momentum. Nature.

[144] Hollingdale MR, Sedegah M. Development of whole sporozoite malaria vaccines. Expert Rev Vaccines. 2016. doi:10.1080/14760584.2016.1203784. 10.1080/14760584.2016.1203784 27327875

[145] Bijker EM, Borrmann S, Kappe SH, Mordmüller B, Sack BK, Khan SM (2015). Novel approaches to whole sporozoite vaccination against malaria. Vaccine.

[146] Heidari R, Shaw DM, Elger BS. CRISPR and the rebirth of synthetic biology. Sci Eng Ethics. 2016. doi:10.1007/s11948-016-9768-z. 10.1007/s11948-016-9768-z 27325413

[147] Pennisi E (2015). Gene drive turns mosquitoes into malaria fighters. Science.

[148] Pearson RD, Amato R, Auburn S, Miotto O, Almagro-Garcia J, Amaratunga C, et al. Genomic analysis of local variation and recent evolution in *Plasmodium vivax*. Nat Genet. 2016. doi:10.1038/ng.3599. 10.1038/ng.3599 PMC4966634 27348299

[149] Hupalo DN, Luo Z, Melnikov A, Sutton PL, Rogov P, Escalante A, et al. Population genomics studies identify signatures of global dispersal and drug resistance in *Plasmodium vivax*. Nat Genet. 2016. doi:10.1038/ng.3588. 10.1038/ng.3588 PMC5347536 27348298

[150] Ghansah A, Amenga-Etego L, Amambua-Ngwa A, Andagalu B, Apinjoh T, Bouyou-Akotet M (2014). Monitoring parasite diversity for malaria elimination in sub-Saharan Africa. Science.

[151] Gulati S, Ekland EH, Ruggles KV, Chan RB, Jayabalasingham B, Zhou B (2015). Profiling the essential nature of lipid metabolism in asexual blood and gametocyte stages of *Plasmodium falciparum*. Cell Host Microbe.

[152] Jones ML, Collins MO, Goulding D, Choudhary JS, Rayner JC (2012). Analysis of protein palmitoylation reveals a pervasive role in *Plasmodium* development and pathogenesis. Cell Host Microbe.

[153] Gomes AR, Bushell E, Schwach F, Girling G, Anar B, Quail MA (2015). A genome-scale vector resource enables high-throughput reverse genetic screening in a malaria parasite. Cell Host Microbe.

[154] Tachibana S, Sullivan SA, Kawai S, Nakamura S, Kim HR, Goto N (2012). *Plasmodium cynomolgi* genome sequences provide insight into *Plasmodium vivax* and the monkey malaria clade. Nat Genet.

[155] Hall N, Karras M, Raine JD, Carlton JM, Kooij TW, Berriman M (2005). A comprehensive survey of the *Plasmodium* life cycle by genomic, transcriptomic, and proteomic analyses. Science.

[156] Carlton JM, Angiuoli SV, Suh BB, Kooij TW, Pertea M, Silva JC (2002). Genome sequence and comparative analysis of the model rodent malaria parasite *Plasmodium yoelii yoelii*. Nature.

[157] Otto TD, Rayner JC, Böhme U, Pain A, Spottiswoode N, Sanders M (2014). Genome sequencing of chimpanzee malaria parasites reveals possible pathways of adaptation to human hosts. Nat Commun.

[158] Sundararaman SA, Plenderleith LJ, Liu W, Loy DE, Learn GH, Li Y (2016). Genomes of cryptic chimpanzee *Plasmodium* species reveal key evolutionary events leading to human malaria. Nat Commun.

[159] Philip N, Waters AP (2015). Conditional degradation of *Plasmodium* calcineurin reveals functions in parasite colonization of both host and vector. Cell Host Microbe.

[160] MalariaGEN Plasmodium falciparum Community Project (2016). Genomic epidemiology of artemisinin resistant malaria. Elife.

[161] Florens L, Washburn MP, Raine JD, Anthony RM, Grainger M, Haynes JD (2002). A proteomic view of the *Plasmodium falciparum* life cycle. Nature.

[162] Lasonder E, Ishihama Y, Andersen JS, Vermunt AM, Pain A, Sauerwein RW (2002). Analysis of the *Plasmodium falciparum* proteome by high-accuracy mass spectrometry. Nature.

[163] Bozdech Z, Llinás M, Pulliam BL, Wong ED, Zhu J, DeRisi JL (2003). The transcriptome of the intraerythrocytic developmental cycle of *Plasmodium falciparum*. PLoS Biol.

[164] Doolan DL, Southwood S, Freilich DA, Sidney J, Graber NL, Shatney L (2003). Identification of *Plasmodium falciparum* antigens by antigenic analysis of genomic and proteomic data. Proc Natl Acad Sci U S A.

[165] Carvalho TG, Thiberge S, Sakamoto H, Menard R (2004). Conditional mutagenesis using site-specific recombination in *Plasmodium berghei*. Proc Natl Acad Sci U S A.

[166] Marti M, Good RT, Rug M, Knuepfer E, Cowman AF (2004). Targeting malaria virulence and remodeling proteins to the host erythrocyte. Science.

[167] Hiller NL, Bhattacharjee S, van Ooij C, Liolios K, Harrison T, Lopez-Estraño C (2004). A host-targeting signal in virulence proteins reveals a secretome in malarial infection. Science.

[168] LaCount DJ, Vignali M, Chettier R, Phansalkar A, Bell R, Hesselberth JR (2005). A protein interaction network of the malaria parasite *Plasmodium falciparum*. Nature.

[169] Armstrong CM, Goldberg DE (2007). An FKBP destabilization domain modulates protein levels in *Plasmodium falciparum*. Nat Methods.

[170] Shock JL, Fischer KF, DeRisi JL (2007). Whole-genome analysis of mRNA decay in *Plasmodium falciparum* reveals a global lengthening of mRNA half-life during the intra-erythrocytic development cycle. Genome Biol.

[171] Salcedo-Amaya AM, van Driel MA, Alako BT, Trelle MB, van den Elzen AM, Cohen AM (2009). Dynamic histone H3 epigenome marking during the intraerythrocytic cycle of *Plasmodium falciparum*. Proc Natl Acad Sci U S A.

[172] Tewari R, Straschil U, Bateman A, Böhme U, Cherevach I, Gong P (2010). The systematic functional analysis of *Plasmodium* protein kinases identifies essential regulators of mosquito transmission. Cell Host Microbe.

[173] van Schaijk BC, Vos MW, Janse CJ, Sauerwein RW, Khan SM (2010). Removal of heterologous sequences from Plasmodium falciparum mutants using FLPe-recombinase. PLoS One.

[174] O’Neill MT, Phuong T, Healer J, Richard D, Cowman AF (2011). Gene deletion from *Plasmodium falciparum* using FLP and Cre recombinases: implications for applied site-specific recombination. Int J Parasitol.

[175] Solyakov L, Halbert J, Alam MM, Semblat JP, Dorin-Semblat D, Reininger L (2011). Global kinomic and phospho-proteomic analyses of the human malaria parasite *Plasmodium falciparum*. Nat Commun.

[176] Straimer J, Lee MCS, Lee AH, Zeitler B, Williams AE, Pearl JR (2012). Site-specific genome editing in *Plasmodium falciparum* using engineered zinc-finger nucleases. Nat Methods.

[177] Pino P, Sebastian S, Kim EA, Bush E, Brochet M, Volkmann K (2012). A tetracycline-repressible transactivator system to study essential genes in malaria parasites. Cell Host Microbe.

[178] Milet J, Sabbagh A, Migot-Nabias F, Luty AJ, Gaye O, Garcia A (2016). Genome-wide association study of antibody responses to *Plasmodium falciparum* candidate vaccine antigens. Genes Immun.

[179] Otto TD, Böhme U, Jackson AP, Hunt M, Franke-Fayard B (2014). Hoeijmakers WA, et al. A comprehensive evaluation of rodent malaria parasite genomes and gene expression. BMC Biol..

[180] Pelle KG, Oh K, Buchholz K, Narasimhan V, Joice R (2015). Milner DA, et al. Transcriptional profiling defines dynamics of parasite tissue sequestration during malaria infection. Genome Med..

[181] Wei C, Xiao T, Zhang P, Wang Z, Chen X, Zhang L (2014). Deep profiling of the novel intermediate-size noncoding RNAs in intraerythrocytic *Plasmodium falciparum*. PLoS One.

[182] Spence PJ, Jarra W, Lévy P, Reid AJ, Chappell L, Brugat T (2013). Vector transmission regulates immune control of *Plasmodium* virulence. Nature.

[183] Ngwa CJ, Scheuermayer M, Mair GR, Kern S, Brügl T, Wirth CC (2013). Changes in the transcriptome of the malaria parasite *Plasmodium falciparum* during the initial phase of transmission from the human to the mosquito. BMC Genomics.

[184] Brochet M, Collins MO, Smith TK, Thompson E, Sebastian S, Volkmann K (2014). Phosphoinositide metabolism links cGMP-dependent protein kinase G to essential Ca^2+^ signals at key decision points in the life cycle of malaria parasites. PLoS Biol.

[185] Alam MM, Solyakov L, Bottrill AR, Flueck C, Siddiqui FA, Singh S (2015). Phosphoproteomics reveals malaria parasite protein kinase G as a signalling hub regulating egress and invasion. Nat Commun.

[186] Tao D, Ubaida-Mohien C, Mathias DK, King JG, Pastrana-Mena R, Tripathi A (2014). Sex-partitioning of the *Plasmodium falciparum* stage V gametocyte proteome provides insight into *falciparum*-specific cell biology. Mol Cell Proteomics.

[187] Guttery DS, Poulin B, Ramaprasad A, Wall RJ, Ferguson DJ, Brady D (2014). Genome-wide functional analysis of *Plasmodium* protein phosphatases reveals key regulators of parasite development and differentiation. Cell Host Microbe.

